# Supplementary material for: Identification of photoperiod-induced specific miRNAs in the adrenal glands of Sunite sheep (Ovis aries)
Source: Front Vet Sci. 2022 Jul 22;9:888207. doi: 10.3389/fvets.2022.888207 (PMC9354845; doi:10.3389/fvets.2022.888207)
Supplement: Supplementary file 1 [file Data_Sheet_1.ZIP › Supplementary file/miRDeep2 file.zip!/miRDeep2 file/Novel_Structure/LP42A3/Novel_40.pdf]

Provisional ID	:	NC_019460.2_5330
Score total	:	184.2
Score for star read(s)	:	3.9
Score for read counts	:	176.5
Score for mfe	:	2.1
Score for randfold	:	1.6
Score for cons. seed	:	
Total read count	:	358
Mature read count	:	290
Loop read count	:	0
Star read count	:	68

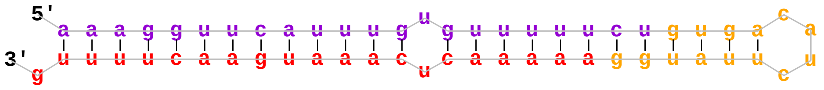

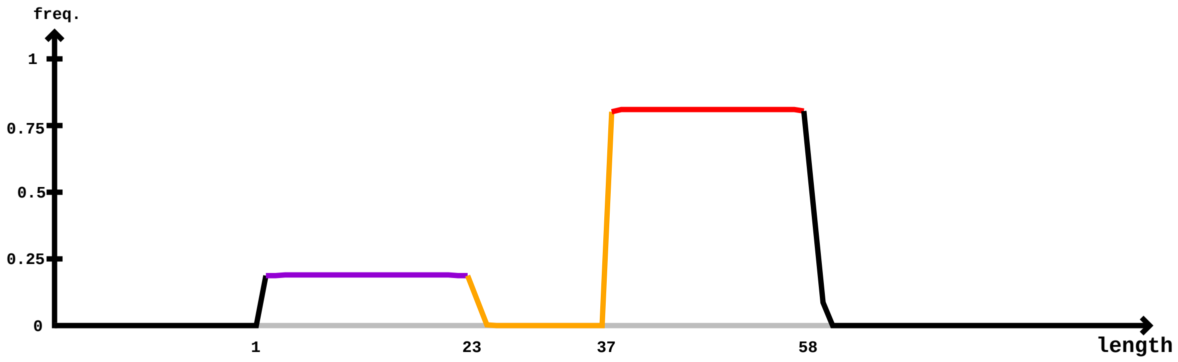

Star **Mature**

```
5'- aaauaauacuggguuggccaaaagguucauuuguguuuuucugugacaucuuaugg aaaaacucaaaugaacuuuugggccaacccaaugaaaagcuccaaucccuucacuc   -3'   obs
    aaauaauacuggguuggccaaaagguucauuuguguuuuucugugacaucuuaugg aaaaacucaaaugaacuuuugggccaacccaaugaaaagcuccaaucccuucacuc         exp
    .........((((((((((.(((((((((((((.((((((((((((....)))))))))))).))))))))))))).)))))))))).........................   reads   mm   sample
    ....................aaagguucauuugGguuuuu........................................................................   1       1    seq
    ....................aaagguucauuuguguuuuucu......................................................................   64      0    seq
    ....................aaagguucauuuguguuuuucug.....................................................................   2       0    seq
    ......................agguucauuuguguuuuucugu....................................................................   1       0    seq
    ........................................................aGaaacucaaaugaacuuuu....................................   1       1    seq
    ........................................................aaaaacucaaaugaacuuuG....................................   1       1    seq
    ........................................................aaaaGcucaaaugaacuuuug...................................   1       1    seq
    ........................................................aaaaacucaaaugaacuuuuA...................................   25      1    seq
    ........................................................aaaaacucaaaugaacuuuug...................................   184     0    seq
    ........................................................aaaaacucaGaugaacuuuug...................................   1       1    seq
    ........................................................aaaaacucaaaugaacCuuug...................................   1       1    seq
    ........................................................aGaaacucaaaugaacuuuug...................................   2       1    seq
    ........................................................aaaaaAucaaaugaacuuuug...................................   1       1    seq
    ........................................................aaaaacucaaaugaacuuuuUg..................................   12      1    seq
    ........................................................aaaaacucaaaugaacuuuugg..................................   30      0    seq
    ........................................................aaaaacucaaaugaacuuuuggU.................................   28      1    seq
    .........................................................aaaacucaaaugaacuuuuUgg.................................   3       1    seq
```
